# Toward a framework for systemic multi-hazard and multi-risk assessment and management

**DOI:** 10.1016/j.isci.2023.106736

**Published:** 2023-04-26

**Authors:** Stefan Hochrainer-Stigler, Robert Trogrlić Šakić, Karina Reiter, Philip J. Ward, Marleen C. de Ruiter, Melanie J. Duncan, Silvia Torresan, Roxana Ciurean, Jaroslav Mysiak, Dana Stuparu, Stefania Gottardo

**Affiliations:** 1 Systemic Risk and Resilience Research Group, International Institute for Applied System Analysis, 2361 Laxenburg, Austria; 2 Institute for Environmental Studies (IVM), Vrije Universiteit Amsterdam, 1081 HV Amsterdam, the Netherlands; 3 British Geological Survey, NG12 5GG Keyworth, EH14 4BA Edinburgh, UK; 4 Euro-Mediterranean Center on Climate Change and Ca’ Foscari University of Venice, Edificio Porta dell’Innovazione - Piano2, Via della Libertà, 12, 30175 Marghera-Venice, VE, Italy; 5 Deltares, 2600 MH Delft, the Netherlands

**Keywords:** Risk assessment, Hazard identification

## Abstract

In our increasingly interconnected world, natural hazards and their impacts spread across geographical, administrative, and sectoral boundaries. Owing to the interrelationships between multi-hazards and socio-economic dimensions, the impacts of these types of events can surmount those of multiple single hazards. The complexities involved in tackling multi-hazards and multi-risks hinder a more holistic and integrative perspective and make it difficult to identify overarching dimensions important for assessment and management purposes. We contribute to this discussion by building on systemic risk research, especially the focus on interconnectedness, and suggest ways forward for an integrated multi-hazard and multi-risk framework that should be beneficial in real-world applications. In this article, we propose a six-step framework for analyzing and managing risk across a spectrum ranging from single-to multi- and systemic risk.

## Graphical abstract

**Figure:**
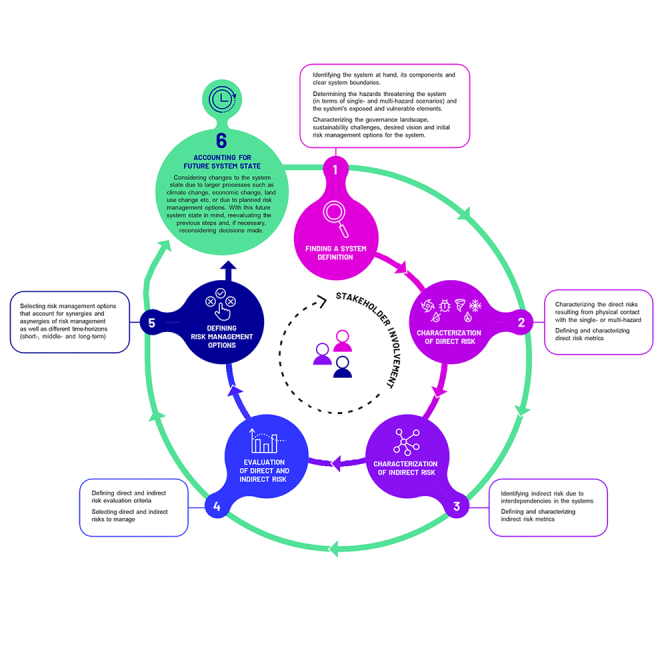

## Introduction

In an increasingly interconnected world, natural hazards and their impacts cascade across geographical, administrative, and sectoral boundaries.^1^^,^^2^ For instance, the Russian heat wave in 2010 happened at the same time as the Indus Valley flooding in Pakistan, which led to a shortfall of cereals in international markets.^1^^,^^3^ More recently, on January 15, 2022, the Hunga Tonga-Hunga Ha’pai volcano erupted near the South Pacific nation of Tonga, resulting in ashfall and triggering a combined pressure wave (far-field) and displacement (near-field) tsunami,^4^ as well as around 70 earthquakes of moment magnitude between 4.4–5.^5^ The eruption generated tsunamis that were observed globally,^6^ reaching the shores of New Zealand, Russia, and causing an oil spill and two casualties in Peru.^5^ The eruption and resulting tsunami happened while the region was impacted by tropical cyclone Cody, which made tsunami detection more difficult because of cyclone-related storm surges.^6^ In the immediate aftermath of the eruption, the situation was compounded by the introduction of COVID-19 in Tonga because of aid arrival through international relief efforts.^4^ The eruption and tsunami resulted in severe impacts with initial direct economic damage of US$90.4 million and projected follow-on multi-sectoral losses and indirect effects in tourism, commercial, agricultural, and infrastructural sectors.^7^

Examples like the above depict the complexities and systemic and far-reaching impacts of multi-hazards and resulting multi-risks and reflect a need for beyond the state-of-the-art assessment and management across different sectors and systems.^2^ In this article, we present a six-step framework for the systemic multi-hazard and multi-risk assessment and management, designed to deal with complex, systemic, and multi-risks. A recent analysis of the biggest challenges in research on natural hazards suggests remaining gaps in understanding multi-hazards and resulting risks.^8^

Rarely is the same geographical location exposed to just a single hazard type; often, a multiplicity of hazards occurs. The United Nations Office for Disaster Risk Reduction (UNDRR) defines multi-hazards as “*the selection of multiple major hazards that the country faces, and the specific contexts where hazardous events may occur simultaneously, cascadingly or cumulatively over time, and taking into account the potential interrelated effect*s”.^9^ Central to this definition and conceptualization of multi-hazard is the fact that multi-hazards refer to multiple single hazards affecting a place and the interrelationships between these hazards. To add complexity to this already challenging problem (and as was indicated by the examples above), these hazards can cause ripple effects and cascading impacts across and between different sectors and systems.^10^

Strong efforts are now made to integrate multi-hazards and multi-risk situations within risk assessment and management methodologies (for a review see Gill et al.^11^) and approaches in practice (for a review see Schlumberger et al.^12^). However, the integration of these methodologies and approaches into a unifying framework, both conceptually as well as for practical applications, is not yet underway.^2^^,^^13^^,^^14^^,^^15^^,^^16^ This comes as no surprise because the complexities involved in tackling multi-hazards and multi-risks eventually hinder a more holistic and integrative perspective and make it difficult to identify some overarching dimensions important for meeting this challenge. This article suggests two overarching dimensions, a specific system definition and the concept of dependencies, and based on these dimensions proposes a possible way forward as to what such a unifying framework could look like.

The definition of a system is critical for determining how to measure improvements when considering various management options.^17^ By clearly establishing the system boundaries, one can identify potential conflicts and avoid the pitfall of narrowly defining the system. For instance, an improvement in a narrowly defined system may not translate into an overall improvement if the boundaries are expanded.^18^ These topics are closely related to systemic risk ideas, where the modeling and measuring of risk are usually done through network dynamics.^19^ Our system definition allows the increase in complexity to necessary levels through a systems-of-systems approach. The additional focus on dependencies, either hazard- or risk-related, enables integration of single, multi-, and systemic risks within the suggested system definition.^20^ Indeed, multi-hazards, as well as multi-risks, can be viewed as single risks in case there are no dependencies. Consequently, single, and systemic risks can be viewed as two ends of a risk continuum where the increase in dependency is the overarching dimension for multi-risks. Based on these ideas, we present a six-step framework for the analysis and management of risk across a spectrum that ranges from single to multi- and systemic risk.

It is beyond the scope of this article to showcase the implementation of this framework in a practical case study (e.g., a region prone to multi-hazard) and this will be done subsequently through pilot studies of the HORIZON 2020 MYRIAD-EU project). This perspective piece rather focuses on the presentation and detailed description of the conceptual framework itself, bringing together research on multi-hazards and multi-risks with research on systemic risks.

The article is structured as follows: Section Muti-hazards, multi-risks, and systemic risk: A brief overview brings an overview of different literature strands which informed the framework development and introduces the expert workshop used in the initial framework refinement. Section Toward a unified framework for individual, multi- and systemic risk assessment and management then introduces in detail each of the six steps of the framework, in parallel introducing a conceptual example. Finally, Section Discussion and conclusions discusses the strengths and limitations of the presented framework and outlines further steps.

## Muti-hazards, multi-risks, and systemic risk: A brief overview

The framework (presented in Section Toward a unified framework for individual, multi- and systemic risk assessment and management) was created drawing on current thinking in multi-hazard and systemic risk literature as well as insights gathered from a feedback workshop with experts. Although it is nearly impossible to give a full overview of current efforts in multi-hazard and multi-risk assessments as well as systemic risk analysis, we have included a selection of recent literature focusing on these topics. Finally, we give a brief description of the stakeholder workshop that informed the framework’s development.

### Multi-hazard approaches and typology

Owing to their interrelationships, multi-hazards might lead to impacts greater than the sum of the effects of individual hazards.^21^ Therefore, multi-hazards should be considered in the assessment and management of disaster risks.^22^ However, current approaches remain focused on single hazards. There is a need for a clear framework for the assessment and management of risks because of multi-hazards^2^^,^^15^ that can integrate multi-hazard approaches into policy, practice, and governance.^23^^,^^24^ There is a growing body of literature on hazard interrelationships^15^^,^^25^^,^^26^^,^^27^^,^^28^^,^^29^^,^^30^ which offers different terms to describe similar interrelationship mechanisms between hazards.^15^^,^^25^ We use the term “interrelationships” as the collective noun for the links. An overview of hazard interrelationships (based on Gill et al.^11^) is provided in the following.

1. **Triggering interrelationship**: One hazard can trigger another hazard to occur. For instance, the 28 September 2018 earthquake at Palu^31^ triggered landslides, or a storm in November 2000 which, in turn, triggered landslides in Tuscany, Italy.^32^ Triggering hazards can result in hazard cascades, chains, or networks when the primary hazard sets off a secondary hazard which then triggers a further hazard.^30^
2. **Amplification interrelationship**: Amplification interrelationships (named “changed condition’’ in Tilloy et al.^25^ or “increasing probability’’ in Gill and Malamud^30^) refers to a situation where one hazard changes the probability or magnitude of another hazard (probability can be both decreased and increased) by changing environmental conditions for the occurrence of another hazard.^29^ A drought can, for instance, increase the probability of a wildfire.^33^
3. **Compound hazards**: A situation in which two or more hazards may impact the same region and/or time period with impacts different (greater, lesser) than their sum.^11^ Compound interrelationships can take different forms: They can, for instance, include interrelationships in which different hazards originate from the same primary event or a large-scale process.^25^ This was the case, for example, for compound coastal floodings in the UK^34^ or compound drought and heat wave events in the Brazilian Pantanal.^35^
Furthermore, they can take the form of a primary hazard simultaneously triggering multiple secondary hazards (e.g., a storm could simultaneously trigger floods and landslides, or a volcanic eruption can produce and trigger multiple hazards to occur at the same time).
Another form of compound interrelationship is that of two independent hazards impacting the same region and/or time period (or in close succession), such as an earthquake followed by a period of extreme cold. These *independent hazards* can occur with no underlying interrelationship between them.^25^ For instance, the 1991 eruption of Mount Pinatubo in the Philippines coincided with Typhoon Yunga.^30^ Recently, there has been an increased interest in *consecutive disasters*, another form of compound hazards. Consecutive disasters refer to a case in which one or more disasters occur after each other and their associated direct impact overlaps in space whereas the recovery from the initial event is still ongoing.^27^ For example, northern Croatia was hit by a 5.5 magnitude earthquake on 22 March 2020,^36^ and then again on 29 December 2020 with a 6.4 magnitude earthquake.^37^ Interactions at the vulnerability level are of importance in consecutive disasters; for instance, Hurricane Matthew in 2016 impacted Haiti which was still in the process of recovery after the catastrophic 2010 earthquake.^29^
The types of hazard interrelationships described above can also overlap in real-life situations, creating complex scenarios.^38^ For instance, in February 2023, earthquakes in Turkey and Syria triggered earthquake aftershocks and landslides,^39^ all while compounded by extreme cold.^40^

It is important to point out that there is no consensus on multi-hazard interrelationships and that many authors provide different definitions and classifications. The three categories described above were identified by summarizing commonalities between hazard interrelationships proposed in the literature.^26^ Furthermore, although hazard interrelationships have recently been receiving more attention, it is worthwhile noting that interrelationships exist at the level of risk and risk components (i.e., between hazard, exposure, and vulnerability), which are important to consider in multi-hazard scenarios and multi-risk assessments.^11^ These interrelationships become prominent already in the description of hazard interrelationships in the examples above (for instance, with compound hazards where hazard interrelationships are described also through impacts). However, the interrelationships on the risk and risk-component side remain understudied.^11^

### Multi-risk approaches and typologies

Over the last decade, there has been increasing interest in introducing multi-hazards in risk assessments. In this article, we adopt the IPCC definition^41^ of disaster risk as a product of hazard (H), exposure (E), and vulnerability (V), whereas disaster risk assessment is defined as “*a qualitative or quantitative approach to determine the nature and extent of disaster risk by analyzing potential hazards and evaluating existing conditions of exposure and vulnerability that together could harm people, property, services, livelihoods and the environment on which they depend”*.^9^

Zschau^42^ provides a classification of risk assessments, distinguishing between four different types thereof.

(1) single-risk: risk in a single-hazard framework;
(2) single-risk: risk in a multilayer single-hazard (i.e., multiple single hazards) framework with no interrelationships on vulnerability level;
(3) multi-hazard risk: risk in a multi-hazard framework (i.e., hazard interrelationships considered) with no interrelationships on the vulnerability level; and
(4) multi-risk: risk in a multi-hazard framework where both interrelationships at hazard and vulnerability levels are considered.

Interrelationships at the vulnerability level (i.e., dynamic vulnerability) refer to vulnerability changes because of different hazards a vulnerable element (e.g., built environment, people) is exposed to over time.^43^ For instance, the vulnerability of a building will be different for floods and earthquakes and can also change in a multi-hazard scenario (e.g., a building hit by a flood after an earthquake).^44^ In their recent article, de Ruiter & van Loon^45^ describe three aspects of the dynamics of vulnerability, including the underlying dynamics of vulnerability (e.g., population immigration and displacement), changes in vulnerability during long-lasting disasters (e.g., droughts), and changes in vulnerability during compounding and consecutive disasters (e.g., a disaster weakening socioeconomic networks).

Although not explicitly referred to in Zschau’s^42^ classifications above, a full multi-risk framework should also consider dynamics of exposure (e.g., people moving to a floodplain following a fire where their exposure to floods is increased). Although it is recognized that Zschau’s^42^ proposed methodology is not easily distinguishable and may require further refinement, it is useful for differentiating between various levels of disaster risk assessments. This holds true especially in the context of the framework proposed in this article, because it is flexible enough to operate on the spectrum of single to multi- and systemic risk assessments by focusing on dependencies (be it hazard, vulnerability, or exposure related) as the overarching concept.

### Multi-risk assessment approaches

Several authors have provided an overview of different approaches for multi-hazard and multi-risk assessments^25^^,^^26^^,^^42^^,^^43^^,^^46^ with detailed methodologies and assessment frameworks available.^15^^,^^47^^,^^48^^,^^49^^,^^50^ Current approaches can usually be classified as either qualitative, semi-quantitative, and quantitative,^42^ applied depending on the research purpose and characteristics of the analysis.^51^ In their review, Ciurean et al.^26^ outline narrative descriptions, hazard wheels, hazard matrices, network diagrams, hazard maps, hazard and risk indices, system-based and physical modeling, and probabilistic and statistical approaches.

Although there have been advances in going from single-risk to full multi-risk assessment frameworks, risks of natural hazards are still primarily considered independently, skewing the decision-making process and management options.^12^^,^^42^^,^^48^ Even in the context of multi-hazards, most risk assessments still primarily address the issue of multi-hazards by overlaying multiple single hazards without considering interrelationships between the hazards.^52^^,^^53^ The challenges associated with the assessment of multi-hazards and multi-risks remain numerous, including a lack of a unified standard and definitions for hazard interrelationships, the inclusion of dynamic vulnerability and exposure, comparability of hazards because of different characteristics, data requirements, levels of complexity, uncertainty in multi-hazard, multi-risk assessments, spatial and temporal dynamics.^15^^,^^21^^,^^42^^,^^46^^,^^51^ The most significant of these challenges is the unavailability of common standards and mature methods for a full multi-risk assessment.^2^^,^^42^^,^^51^

To address the above-mentioned challenges to a certain extent, the framework presented in this article proposes using the concept of dependency (or more broadly: connectedness) as a unifying approach that can incorporate both single and multi-hazard as well as risk approaches. By focusing on dependencies, the framework provides a possible way forward to overcome the current lack of common standards and methods for conducting full multi-risk assessments.

### Systemic risk approaches

A comprehensive literature review of systemic risk in the context of natural hazards can be found in Hochrainer-Stigler.^54^ Systemic risk refers to how subtle changes within a system may trigger the collapse of the system itself. The primary mechanism usually looked at in that regard is that of contagious risks, i.e., risks that can spread from one element to another and therefore cause cascading and possible negative feedback effects.

After the financial crisis in 2007/08, a lot of research on systemic risk focused on banking and financial systems (for a detailed historic analysis we suggest Kreis et al.^55^) and found that the most important specific mechanisms that can cause failure include *too big to fail*, *too interconnected to fail* and *keystone elements*. Hence, the failure of a system element as well as the dependency structure within the system in regard to the element is a cornerstone of systemic risk analysis, probably the most important measure now used in that regard being DebtRank^56^ which can also be used for policy analysis.^57^

Social sciences have also contributed significantly to systemic risk analysis, exploring how systems are exposed to systemic risk and identifying the distinct features of such systems^58^ However, the missing link between the natural science approaches and the social sciences, as identified by Hochrainer-Stigler et al.,^59^ is the human agency aspect, which needs to be included for systemic and complex adaptive systems research. For a more pragmatic approach to systemic risk analysis within real-world decision-making processes, we refer to Sillman et al.^16^

### Workshop for gathering feedback and informing the development of the framework

In addition to building the framework based on the existing multi-hazard and multi-risk thinking and frameworks as outlined above, the framework was also informed by a workshop held with scientific and practitioner experts in April 2022. The overall aim of the workshop was to present the prototype framework developed based on the extensive literature review and to have an informed discussion with participants and collect their critical reflections.

The workshop had a total of 62 participants, including representatives from the HORIZON 2020 MYRIAD-EU consortium partners (n = 37), external experts in the field of multi-risk (n = 17), case study pilot representatives, and wider sectoral representatives (n = 8). External experts were identified in a process of consultation with researchers and represented a mix of academic researchers, representatives of multilateral organizations (e.g., World Bank, United Nations Office for Disaster Risk Reduction), and MYRIAD-EU pilot stakeholders. The workshop was interactive and held in a hybrid format, with roughly half of the participants joining in person, whereas the other half participated online. It consisted of plenary lectures and discussions as well as interactive discussions in smaller groups.

During the workshop, participants were presented with the prototype version of the framework (see Figure S1), followed by a detailed explanation. Furthermore, before the workshop, a brief description of the framework was shared with participants. The prototype framework was developed by a team of MYRIAD-EU researchers (from September 2021 to March 2022) and built on existing multi-risk and systemic risk assessment approaches and typologies (described in Section Multi-hazard approaches and typology, Multi-risk approaches and typologies, Multi-risk assessment approaches, and Systemic risk approaches). During the workshop, participants were asked to discuss the strengths and weaknesses of the framework, identify what the framework was lacking, and provide suggestions for improving framework. By focusing on these questions and careful facilitation of discussions, every effort was made to ensure that the feedback from the discussions was steered toward a critical reflection of the framework.

The workshop feedback indicated that the framework has a clear structure and a stepwise approach that was appreciated. However, participants suggested emphasizing further the interconnections between the different steps of the framework. As a result, the framework was converted from a linear stepwise procedure (see Figure S1) to a circular model. Participants also emphasized the benefits of a framework that is flexible to accommodate a continuum from individual to multi- and systemic risk analysis, the inclusion of direct and indirect risks, stakeholder engagement, and the relevance of findings to policy and decision-making. Regarding the required changes, several issues were raised during discussions that were incorporated into the updated version of the framework presented in this article. These issues included:

• Improving the consistency of language within the framework to make sure that various terms are used in the same manner throughout the graphic and accompanying text.
• Including a clear statement of challenges for the system and desired state in Step 1, as well as a mapping of policies, institutions, and stakeholders.
• Already including discussions on possible risk management options in Step 1.
• Emphasizing the interaction between the different steps, resulting in a circular representation of the framework.
• Changing the language in Step 6 to make it more accommodating (e.g., urban growth vs. urban change, economic growth vs. economic change).
• Aligning the framework with the stages of the risk assessment process as outlined in Poljansek et al.^60^

In Section Toward a unified framework for individual, multi- and systemic risk assessment and management, we present the framework that incorporates this feedback.

## Toward a unified framework for individual, multi- and systemic risk assessment and management

**Figure 1:**
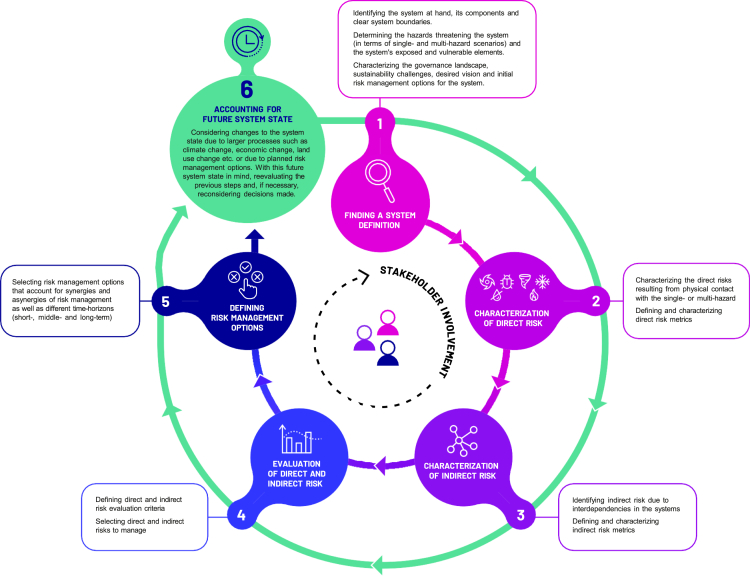
Six-step framework for individual, multi-, and systemic risk analysis and management

The Merriam-Webster Dictionary defines the term framework as a “*basic conceptual structure (as of ideas)*”. Similarly, the Oxford Dictionary defines a framework as “*a set of beliefs, ideas or rules that is used as a basis for making judgments, decisions, etc.*”. In this article, building on these definitions, we define a framework as “*a frame one can work with*”. The framework is developed to be useful for practical application and, therefore, follows a pragmatic approach. It is a stepwise and iterative process comprising six major steps as presented in Figure 1. In what follows, each of its steps will be discussed through a conceptual example including a thorough discussion of the ideas behind each step.

### Step 1: Finding a system definition

At the very start of the framework, one needs to understand the system under consideration. This means that one must delineate clear system boundaries and identify the elements of the system that lie within these boundaries. In other words, identifying the system boundaries makes it possible to clearly identify the system elements and answer the question of which elements lie within and outside the system. Who defines the system boundaries and for what reason(s) is an important question that needs to be addressed, not least to indicate conflicts between potential risk bearers and interactions that may or may not (yet) be incorporated in the analysis. More generally speaking, one must assume that perceptions will differ as to which system elements or sub-systems are important within the system at the conceptual level and which can be identified at the practical level. Furthermore, divergent views of decision-makers need to be assumed as well; this can refer to diverging views on the system boundaries and, as a result, the systems elements as well as the interdependencies between these elements. Important to note is that system boundaries can overlap between different system owners (i.e., what is in the system defined by stakeholder A can also be in the system defined by stakeholder B). In addition, interventions might entail profound changes for certain stakeholders within the system and its sub-system as well as across interconnected and interdependent systems; for others, the changes might be minimal. Explicitly defining the system boundaries and its internal complex networks of interdependencies is needed for tackling these challenges.

**Figure 2:**
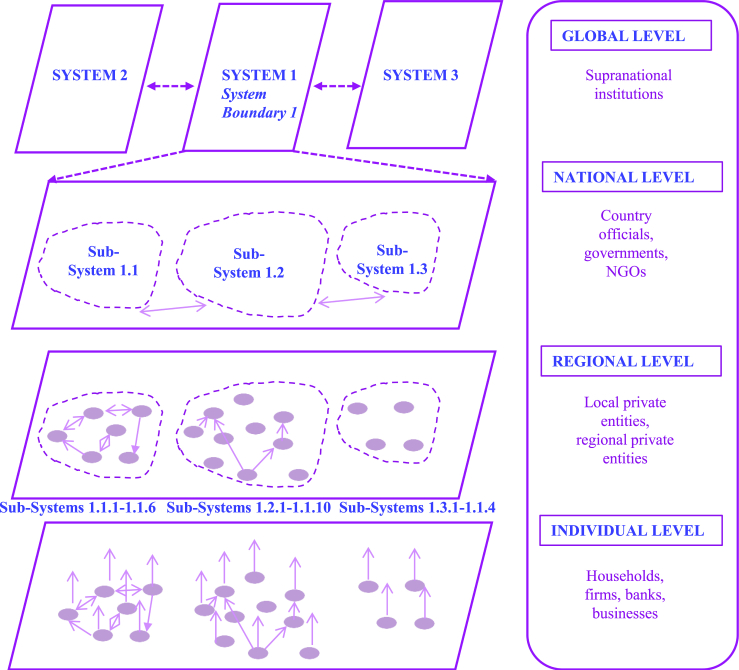
A system of systems approach using system boundaries as an overarching principle On the lowest level, only individual elements are considered which can form sub-systems on higher levels. Sub-System 1.1. consists of 6 individual elements, Sub-System 1.2. consists of 10 individual elements and Sub-system 1.3 consists of 4 elements, System 1 either consists of 3 elements or sub-systems or 20 individual elements. As depicted in the figure, there can be interdependencies between systems and sub-systems (represented by the arrows connecting different elements of the system).

**Figure 3:**
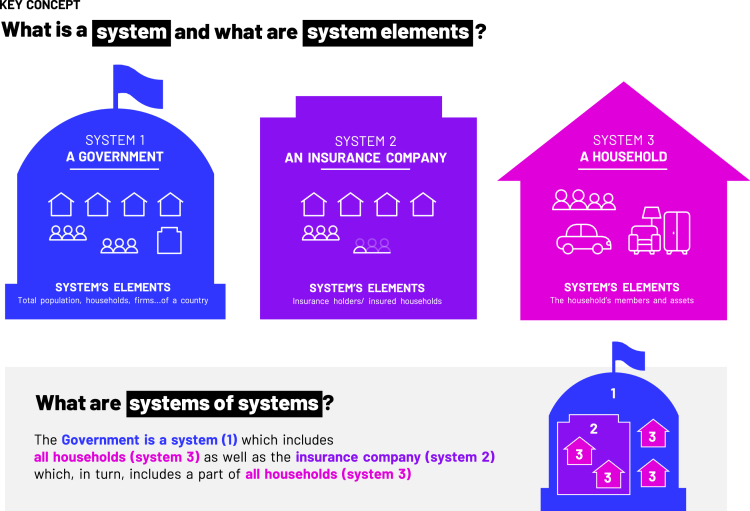
Graphical representation of key concepts used in the article (i.e., system, system elements, and system of systems) explained using a conceptual example of a government, an insurance company, and a household

**Figure 4:**
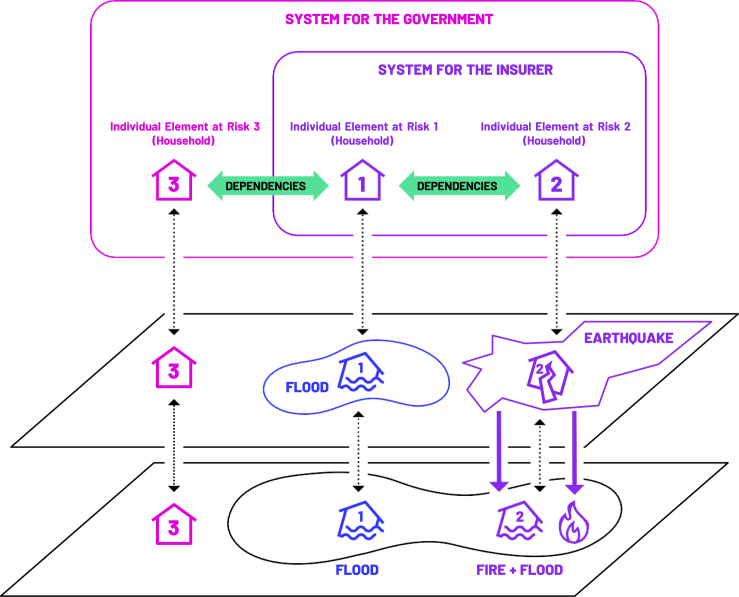
Simple example of three individual elements at risk under a systemic perspective (i.e., with interdependencies between elements of the system)

In this article, we define a system *as a set of (partly) interconnected elements with clear boundaries*. In addition, we define system of systems *as a system in which its elements can again be seen as systems*. Figure 2 introduces an example of a system and system elements and shows a possible system of systems approach based on different geographical scales and possible actors involved. These key concepts, i.e., systems, systems elements, and systems of systems, are depicted in Figure 3. To provide a clearer understanding of the framework, Figure 2 illustrates the differentiation between elements on different system levels. To further demonstrate the application of the framework, we present a conceptual example in Figures 3 and 4, which includes households, an insurance provider, and the government as possible systems.

Another crucial step in understanding systems is identifying current and/or future challenges for the system holder (e.g., the government, a specific sector, etc.) through engaging with a specific sustainability challenge (e.g., resilience to multi-hazards of interconnected countries with strong macroeconomic relationships, disaster resilience of islands with strong economic dependence on tourism, etc.).^2^

Given that the framework is focused on natural-hazard related risks, a system definition also includes the identification of hazards of interest and associated hazard scenarios (including both single and multi-hazards), as well as the identification of exposed and vulnerable elements of the system (e.g., assets and people located in hazard-prone areas together with different dimensions of vulnerability, such as economic, social, institutional, physical, etc.). Different natural hazards of interest can be identified together with their interrelationships^15^^,^^25^^,^^26^^,^^30^ (as presented in Section Muti-hazards, multi-risks, and systemic risk: A brief overview).

A system definition also requires an understanding of the existing governance landscape (i.e., main policies, institutions, and stakeholders guiding the system), including understanding the gaps that contribute to the sustainability challenges identified. This can help in, for instance, defining what a desired outcome for the system is as defined in a specific policy (e.g., sectoral policies, disaster risk management policies). Finally, system definitions help take stock of the existing risk management options (e.g., different structural and non-structural measures) and identify potential gaps in this management landscape.

### Step 2: Characterization of direct risk

The framework defined in this article uses the IPCC definition of risk, which focuses on hazard, exposure, and vulnerability.^41^ Direct risk is related to losses because of direct contact of system elements with the single or multi-hazard itself. Determining direct risk means selecting direct risk metrics as a measure of risk. These should be set by engaging with stakeholders who can give insights as to which metrics are most important for them.^61^ For direct risk metrics, a variety of options is available, such as physical asset losses, casualties and the proportion of the population experiencing monetary loss because of their assets being hit by a hazard (*ibid*.). As presented by Poljansek et al.,^60^ risk metrics are essential tools for decision-making and engaging with stakeholders in disaster risk management. In this step, the changing nature of exposure and vulnerability in a multi-hazard scenario also needs to be considered.^27^^,^^45^^,^^62^^,^^63^

A description and terminology of multi-hazards and multi-risks are given in Section Muti-hazards, multi-risks, and systemic risk: A brief overview, including a discussion of similarities and differences. As one can see, the hazards, possible interrelated events, and interactions in terms of drivers and processes (may it be climate-related or of socio-economic nature) can be conceptually integrated using the concept of dependency (or more broadly, connectedness), which ranges from independency to full dependency (such as within physical laws).

To avoid confusion, we now introduce a conceptual example as presented in Figure 4, starting with the simplest case, i.e., that of an individual exposed to some risk. We will call this “Individual Element 1 at Risk”. As our focus is on natural hazard events, we further assume that this risk is pure downside risk, i.e., risk realized only in the form of losses. We then introduce an additional element, which we call “Individual Element 2 at Risk”.

Each element can assess its own risk and perform a risk analysis to inform risk management decisions. However, we may also want to consider both individual risks simultaneously. When looking at Element at Risk 1 and Element at Risk 2 simultaneously, one can introduce the term “system” as *a set of (partly) interconnected elements*. Furthermore, we define the term “individual risk” as *the risk an individual element is exposed to inside the system* whereas “systemic risk” is *the risk on the system level because of the dependencies of the elements inside the system*. To add some additional complexity, there might also be an “Individual Element at Risk 3”. If we have not introduced this element (or if it is of no interest to the risk bearer of the system), the given system just consists of the two individual elements at risk (as presented in Figure 4) in the first instance. This highlights the need for clear boundaries when defining the system under study, which is a crucial aspect of our framework as discussed in Step 1. In other words, the system needs to be defined in terms of which elements are inside the system and which are outside of it.

In our example, Household 1 and Household 2 are the system elements of the insurer whereas Households 1, 2, and 3 are the system elements of the government. For direct risk, only Household 1 and Household 2 are of interest to the insurance provider. Furthermore, for Household 1 only a single risk (flooding) is of interest. Meanwhile, multiple hazards are relevant for Household 2 because of the interaction of an earthquake that increased the flood area so that Household 2 is affected by flooding as well as fire. Consequently, these hazards are also important for the insurance provider.

### Step 3: Characterization of indirect risk

Indirect risk refers to risk realized because of interdependencies within the system. These interdependencies can exist between the elements within the system or between systems themselves, such as sub-systems within a larger system, as shown in Figures 2 and 3 (it is important to note that elements of the system can be systems themselves). We consider indirect risk only through the lens of losses that occur because of direct risks. These can take the form of, for instance, losses in the agricultural sector because of direct damages to transport infrastructure, which, in turn, influence agricultural supply chains. Indirect losses can occur either inside or outside of the area hit by a hazard and often with a time lag.^64^ In line with the systems perspective, this means that these losses propagate across and beyond system boundaries. In this step, indirect risk metrics are selected and agreed on in collaboration with stakeholders. Example metrics include the costs of disrupted supply chains or a decrease in purchasing power or more general systemic risk measures (see for a review Hochrainer-Stigler et al.^59^).

The ideas in Step 3 are grounded in systemic risk research. There, systemic risk is usually defined as a serious disruption or collapse of a system. With standard application in other contexts (e.g., financial systems), the concept of systemic risk and its analysis and management is gaining rising traction in research on disaster risk reduction and climate change.^65^ Systemic risks challenge the conventional approach to risk analysis and management.^66^ This is because of inherent characteristics of systemic risk.^67^ For instance, Renn^66^ identifies four major components of systemic risk, namely: (1) Complexity, (2) uncertainty, (3) ambiguity and (4) ripple effects beyond the source of risk. Because of these characteristics, systemic risks challenge and overburden existing risk management and create new challenges for risk assessments, policy making, and governance.^58^

The defining feature of systemic risk is the concept of interdependencies within the elements of the system (also called feedback loops, interactions, interconnections, interlinkages, and intertwined elements^16^). As discussed in our conceptual example where there is an absence of interdependencies, one can refer to risks to the individual elements in the system as individual risks. These risks exist because of individual events that have a direct impact on the element in the system, independently from the rest of the system.^20^ However, failures of the individual elements in the system may trigger multi-risks and systemic risks and therefore, individual, compound, multi- and systemic risk can and should be assessed and managed together.^20^^,^^67^ Which of these processes and risks dominate is determined by the distinct dependencies of the system and its elements at hand. Although there are a number of emerging methods for systemic risk analysis, including copula-based approaches^67^ and agent-based modeling,^68^ there is a need for an integrative and holistic approach allowing for analytical perspectives based on a variety of data (e.g., observational, experimental, simulations, quantitative, and qualitative) as well as the specific aspects of human agency as we proposing in the framework herein.^16^^,^^58^^,^^59^^,^^66^

**Figure 5:**
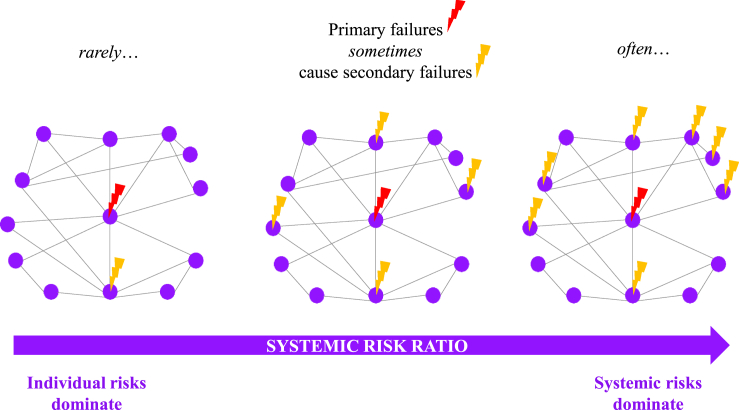
The continuum between individual risks and systemic risks System components (purple circles) interact (black lines) in a networked system (e.g., hazards, sectors, time). Owing to these interactions, primary failures (red flashes) can trigger secondary failures/events (orange flashes). The systemic-risk ratio (purple arrow) measures the proportion of all secondary failures (Adapted from Hochrainer-Stigler et al.^20^).

The concept of dependency opens a promising way forward to simultaneously include single, compound, and multi-risk as well as systemic risk within one unifying framework. We suggest that the dependency between the elements of a system can function as a guiding principle here. Figure 5 illustrates the risk continuum that arises from different levels of dependency which range from individual risk, multi-to systemic risks (in the classic sense of full failure of the system, systemic risk as used here has a broader notion). Viewed from a systemic perspective and using our system definition which includes a system of systems approach, the so-called failures of elements can be reinterpreted as events that cause consequences because of dependencies. The stronger the dependencies are, the more the system level will be affected, e.g., systemic risk dominates. If the dependencies between the system elements is weak (i.e., if the system elements are independent of one another), individual risks dominate.

What kind of dependencies can occur in systems is not discussed yet and is highly context-specific. However, the amount and strength of dependencies between the elements in a system can be used to separate strategies from a top-down perspective as well as a bottom-up perspective which have quite different instruments at hand. As depicted in Figure 5, in the case that individual events (e.g., hazards) do not cause failures, these can be seen as individual risks (left hand of the systemic risk ratio in Figure 5). On the opposite end, as primary failures often cause secondary failures (or cascades), systemic risks may dominate. It is important to note that dependencies of the elements within a given system may also change depending on the hazard impact (e.g., very large losses) or on resources available to deal with losses (e.g., financial ones such as savings or having insurance or not) (see Hochrainer-Stigler et al.^67^).

Within our conceptual example with a household, insurer, and the government as shown in Figure 4, Household 3 is not directly affected by natural hazard events. However, it is indirectly affected by hazards because of its dependency on Household 1 (e.g., economic dependencies). As a result, the government also faces indirect risks (e.g., because of unemployment and corresponding costs, or a decrease in tax revenue because of a decrease in purchasing power) in addition to the direct risks to which Household 1 and Household 2 are exposed (for which the government might have to provide disaster relief). Note that in our example the indirect risk for Household 3 is only the result of a single hazard event (flooding) that happens within the region where Household 1 is located. However, in the case of a multi-hazard event (e.g., flood and earthquakes), the flood event may increase and could be more devastating (e.g., because of amplification effects, see methods section) even if it were only a single hazard event for Household 1. In addition, also the indirect risk could increase because of limited resources to cope with the event (for a modeling example we refer to Hochrainer-Stigler et al.,^67^ for a concrete example of the European Solidarity Fund we refer to Ciullo et al.^69^). In other words, Household 1 is not only directly exposed to the natural hazard event, but also indirectly exposed to additional risks because of its dependency on Household 2. This dependency creates indirect risks for Household 1 that could also affect Household 3.

### Step 4: Evaluation of direct and indirect risk

In alignment with the stages of the risk assessment process according to ISO31010, Poljansek et al.^60^ distinguish between risk identification, risk analysis, and risk evaluation. Although we dealt with risk identification in Step 1 and risk analysis in Step 2 and Step 3, the next stage in our framework is risk evaluation. The purpose of risk evaluation is to support decision-making.^60^

Based on the risk metrics used within the risk analysis steps (for a summary of measures for the direct and indirect risk we refer to Hochrainer-Stigler et al.^20^), decision-makers need to decide if the risks at hand are acceptable or if they need to be managed. For some decision-makers, this may be easier to determine than for others. Depending on the system level, the measures may be quantifiable or not and may depend on the policy landscape they are embedded in. For example, insurance companies will need to look only at direct risks, e.g., using a loss distribution approach that can be used to determine backup capital needed with regard to regulation requirements.^70^ A government may base its decision on how much resource it has to finance direct losses based on the resource gap concept^71^ and how to reduce indirect losses in case it is not able to finance all direct losses.^72^ A household may decide based on savings and insurance availability as well as assistance from the government. The decision may, however, also include intangible dimensions such as environmental ones (see Hudson et al.^73^ for discussion).

### Step 5: Risk management options

In this step, risk management options are discussed and decided on based on risk evaluation, initial discussions in Step 1, and the results of the direct and indirect risk assessment. There is a wide range of available options for risk management including structural (e.g., structural defenses) and non-structural measures (e.g., policies, land zoning, early warning systems).^9^ Similarly, the Society of Risk Analysis (2015) suggests three types of risk management options, namely risk-informed strategies, precautionary strategies, and discursive strategies.^74^^,^^75^ There will always be a mix of diverse types of strategies and measures at decision makers’ hands. Risk management options need to be selected in collaboration with stakeholders depending on, for instance, the risk metrics agreed on in a decision forum^61^^,^^76^ with a wide range of decision support tools available. In addition, risk management measures should be considered for different time horizons and planning periods, from the short-to mid-and long-term. In a multi-risk context, the process of selecting risk management measures also needs to pay attention to synergies and trade-offs (i.e., asynergies) between risk management options for different hazards.^44^^,^^77^

**Figure 6:**
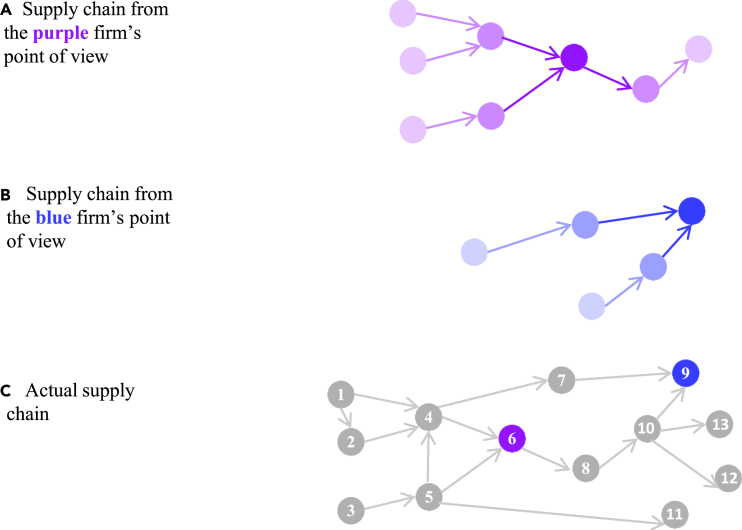
Schematic diagram of a supply chain perceived by two firms (A and B) and how it actually is (C) Each bubble represents a firm, and each arrow represents a supplier–buyer relationship. The blue (A) and purple (B) firms share the same supply chain, but their perspective on it is different. One can assume that firms know their tier-two suppliers and clients. The lighter the color of the supplier, the clients, and the linkages, the less information the firm has on them. In panel (C), the entire supply chain is represented and firms are identified by numbers. Source: Colon and Hochrainer-Stigler.^78^

The system definition as well as dependency concept can be used as a guiding principle between bottom-up and top-down management approaches. The former focus on risk reduction for individual elements whereas the latter focus on managing the dependencies between the system elements. Both usually have quite different measures at hand because their expertise differs too. Supply chain risk management, for example, is usually done on the firm level, and each firm assesses and manages its risks only with respect to its most relevant suppliers. It, therefore, could be interpreted as a bottom-up approach, e.g., the elements of the system are managing their risk. However, the firms are embedded in a more interdependent system as they are aware of and may be affected by other suppliers. These interactions have to be taken into account by a systemic perspective, i.e., top-down; for instance, setting up regulations to reduce systemic risks^78^ (Figure 6).

In general, local-level decision-making processes may not be sufficient to address systemic issues, whereas systemic-level decision-making processes may not be aware of or able to effectively utilize the options available at the local level. Nevertheless, both are intrinsically related and, therefore, need to be looked at together.

In our conceptual example, the government (top-down approach) may focus on the multi-hazard risks and build dikes to avoid losses for Household 1. As a result, the indirect risk to Household 3 is also mitigated. By constructing dikes, the government could limit the extent of a flood event and prevent it from affecting Household 2, which would also reduce indirect risk. In this way, the government can change the hazard dependencies and subsequently, both the direct and indirect risks. The government could also support the insurance company through subsidies so that the insurance provides insurance schemes in the region where Households 1 and 2 are located so that indirect risk may not spread to Household 3 (e.g., through economic dependencies, see the supply chain risk example in Figure 6). This approach would focus more on socio-economic dependencies rather than hazard dependencies. Other options focusing on local-level risk reduction would be also possible (this would be a bottom-up approach) and could be combined with the top-down approach as well (e.g., giving subsidies for risk reduction).

### Step 6: Future system state

Step 6 of the framework addresses the question of how to maneuver through an uncertain world (e.g., Schlumberger et al.^79^). Given the projected changes in risk components (i.e., hazard, exposure, and vulnerability) because of a number of processes, it is of critical importance to consider risk management in the context of these future changes to be able to take risk-informed decisions that will allow for reduced risks in the future.^80^ The framework, therefore, has to be iterative and must allow its user to consider future changes in the system and how these could influence individual, multi- and systemic risk. This step considers changes to risk components because of larger processes (e.g., climate, demographic, political, and land use change) as well as because of changes in the system because of risk management options implemented (e.g., risk management is more recently discussed as one of the risk components in Simpson et al.^81^). Given the effects of these processes, the system itself will change (e.g., the number or condition of the elements at risk or the system boundaries might change) and the direct and indirect risks it is exposed to need to be reevaluated. In addition, one needs to consider how proposed risk management options will perform in the future system state and make adjustments accordingly. Existing methodologies for decision-making under uncertainty play an important role in this process.^79^^,^^82^

### Importance of stakeholder engagement and co-production

The involvement of different stakeholders is integral throughout all the steps. For instance, the definition of system boundaries and multi-hazard scenarios of interest will vary between different stakeholders (e.g., stakeholders in the tourism sector versus an insurance company, stakeholders ranging from local to regional and global scales). Furthermore, the direct and indirect risk metrics (i.e., which risk metric is most relevant for a specific sector), risk evaluation criteria, as well as risk management options, should be co-developed with stakeholders.^61^^,^^76^^,^^83^

## Discussion and conclusions

Given the rising importance of accounting for multi- and systemic risk in disaster risk management^8^^,^^15^^,^^27^^,^^30^ and the lack of a unifying framework to guide the analysis of these risks,^2^^,^^20^ we suggested a six-step framework based on two overarching dimensions–a specific system definition and the concept of dependency. The framework itself overcomes some of the limitations of the existing frameworks for multi-risk assessment because of the following.

• **Flexibility to address single-to multi- and systemic risk:** The framework is based on a system dependency perspective meaning that, based on the level of interdependencies between system elements and different systems (system-of-systems perspective), it can be used for the analysis of individual, multi- and systemic risk, which cuts across all steps of the framework. This way, it can accommodate different existing tools and methods, and levels of analysis. In addition, it can be tailored according to the context (i.e., the system of interest) and in line with stakeholder needs. Existing frameworks for multi-risk assessment are primarily based on one specific method (e.g., Liu et al.^47^ use Bayesian networks).
• **Account for risk dynamics:** The framework considers risk and all its components (i.e., hazard, exposure, and vulnerability) to be inherently dynamic through (1) the dynamics of exposure and vulnerability in a multi-hazard scenario, (2) changes of risk through different socio-economic interaction channels (e.g., economy), (3) changes in the system considered in Step 6 that directly affect the hazard, exposure, or vulnerability. Existing frameworks often omit these dynamics (e.g., Schmidt et al.^48^) or consider them only partially (e.g., Mignan et al.^49^ account only for changes in the structural vulnerability of buildings).
• **Explicit focus on indirect risk:** By focusing on dependencies and a systemic risk perspective, the framework places and explicitly focuses on indirect risk, which is particularly important in the context of cross-boundary and cross-sectoral risks.^10^^,^^72^ Other available frameworks either do not consider indirect risks (e.g., Liu et al.^47^ focus on direct risks for buildings) or do not place such an explicit focus on risks that arise because of interdependencies between system elements (e.g., De Angeli et al.^15^ acknowledge the importance of indirect risk but do not provide an explicit focus).
• **Multiple-line of evidence approach**: The proposed framework allows and asks for the integration and use of different types of data, from a qualitative and narrative implementation of the framework (e.g., the framework could be implemented through a workshop-style activity with stakeholders that would engage around the topic of multi-risk management) to a comprehensive quantitative risk assessment (e.g., quantification of interactions between different hazards). The explicit request for stakeholder engagement warrants the inclusion of qualitative data and a focus on co-production. Most existing frameworks are based only on quantitative methods which are primarily of a probabilistic nature (e.g., Liu et al.^28^; Marzocchi et al.^50^) whereas a similar approach to the integration of both qualitative and quantitative data was proposed by Liu et al.^47^
• **System of systems perspective allowing for risk analysis and management across scales**: By taking a systems perspective and asking for a clear delineation of system boundaries, the framework enables systems at different levels to be viewed from a systems perspective. As a result, it facilitates risk management at different levels and the identification of risk management options at both the local level (i.e., bottom-up) and the system level (i.e., top-down). This is helpful in terms of risk governance, clearly defining responsibilities for risk management options at different levels and considering synergies and asynergies between risk management actions. Previous frameworks lack this perspective, making it challenging to determine which management options work best at what scale and under whose responsibility.
• **Strong emphasis on stakeholder engagement and co-production**: In the framework, various types of stakeholders (e.g., local communities through to different levels of government to regional and global agencies - depending on the level of analysis) are actively involved and shape different steps of the framework (through, for instance, determining system boundaries and identifying risk metrics). Many existing frameworks do not explicitly take into account stakeholder input in the process of framework implementation e.g., (De Angeli et al.^15^; Liu et al.^28^^,^^29^; Schmidt et al.^48^; Simpson et al.^81^), whereas consultation with stakeholders is envisioned in Liu et al.^47^ and Marzocchi et al.^50^ Framework implementation in practice should be done through co-production with various stakeholders, where different perspectives should be explicitly taken into account, including local knowledge of communities, as this knowledge is crucial in designing context-appropriate risk management strategies.^84^ Special emphasis will need to be given to creating stakeholder engagement practices to counter conflicting and contested stakeholder objectives.^79^ The concept of risk democratization introduced by Cremen et al.^76^ could be a useful approach to aid this process.
• **Forward-looking and embedded in larger sustainability issues**: The framework starts with the identification of sustainability challenges in the system at hand, enabling the identification of forward-looking disaster risk management pathways. By starting from a sustainability challenge, it also goes beyond simply viewing natural hazard risks through a hazard-oriented lens but takes a risk-informed approach and enables hazards to be considered in the context of sustainability challenges (e.g., risk-informed urban development as outlined in Galasso et al.^85^). By considering the future state of the system (Step 6), the framework also explicitly considers future risks arising from (1) larger processes such as climate change and land use change, and (2) adoption of risk management measures (Step 5).

Finally, there are also some limitations including the fact that the framework is complex and requires in-depth technical knowledge to implement the various steps. However, there is the question of how much one can reduce complexity to manageable levels while not missing some essential characteristics of the system for its successful management. As discussed, single hazard and risk approaches are ill-equipped today for meeting compounding challenges ahead. Therefore, much more research needs to go into the analysis of interdependencies within systems and risks in the future. Furthermore, implementing the framework potentially requires a large amount of data, especially in terms of quantitative analysis, which may not be readily available. As a possible way forward, scenario approaches and storylines^86^ as well as adaptation pathways^79^^,^^82^ may be a good first step in reducing data requirements and complexity to manageable levels. The role of “optimal complexity” will eventually be an important research agenda in that regard.

The framework presented herein can be applied by different types of stakeholders operating across different scales of disaster risk landscapes (e.g., from local to global levels), owing to its explicit consideration of system perspective and a strong emphasis on stakeholder engagement. For instance, it can be a useful tool for government departments dealing with disaster risk to analyze multi-risks at different spatial scales (e.g., city, regional, or national scale) in a given country and guide their decision-making on risk management options. Similarly, it could be applied by an insurance company to analyze the risks of their premium holders in a given region, as described in a conceptual example. In both cases, a clear system definition presents a critical step.

Although the framework is based on ideas from systemic risk research in conjunction with literature reviews and stakeholder interactions including high-level workshops (see Section Muti-hazards, multi-risks, and systemic risk: A brief overview), it has not been tested in real-world applications. It will, however, be implemented in five pilot case study areas in Europe over the next 3 years (Scandinavia, Danube Region, Veneto Region, North Sea, and Canary Islands). Nevertheless, the framework should already be beneficial in current efforts that tackle the governance and modeling challenges regarding multi-risk by suggesting focusing on system boundaries and dependency dimensions.

## Data availability

This article did not use any primary data.
