# Supplementary material for: Toward a framework for systemic multi-hazard and multi-risk assessment and management
Source: iScience. 2023 Apr 26;26(5):106736. doi: 10.1016/j.isci.2023.106736 (PMC10196580; doi:10.1016/j.isci.2023.106736)
Supplement: Document S1. Figure S1 [file mmc1.pdf]

iScience, Volume *26*

Supplemental information

Toward a framework for systemic multi-hazard and multi-risk assessment and management

Stefan Hochrainer-Stigler, Robert Trogrlić Šakić, Karina Reiter, Philip J. Ward, Marleen C. de Ruiter, Melanie J. Duncan, Silvia Torresan, Roxana Ciurean, Jaroslav Mysiak, Dana Stuparu, and Stefania Gottardo

Supplementary material

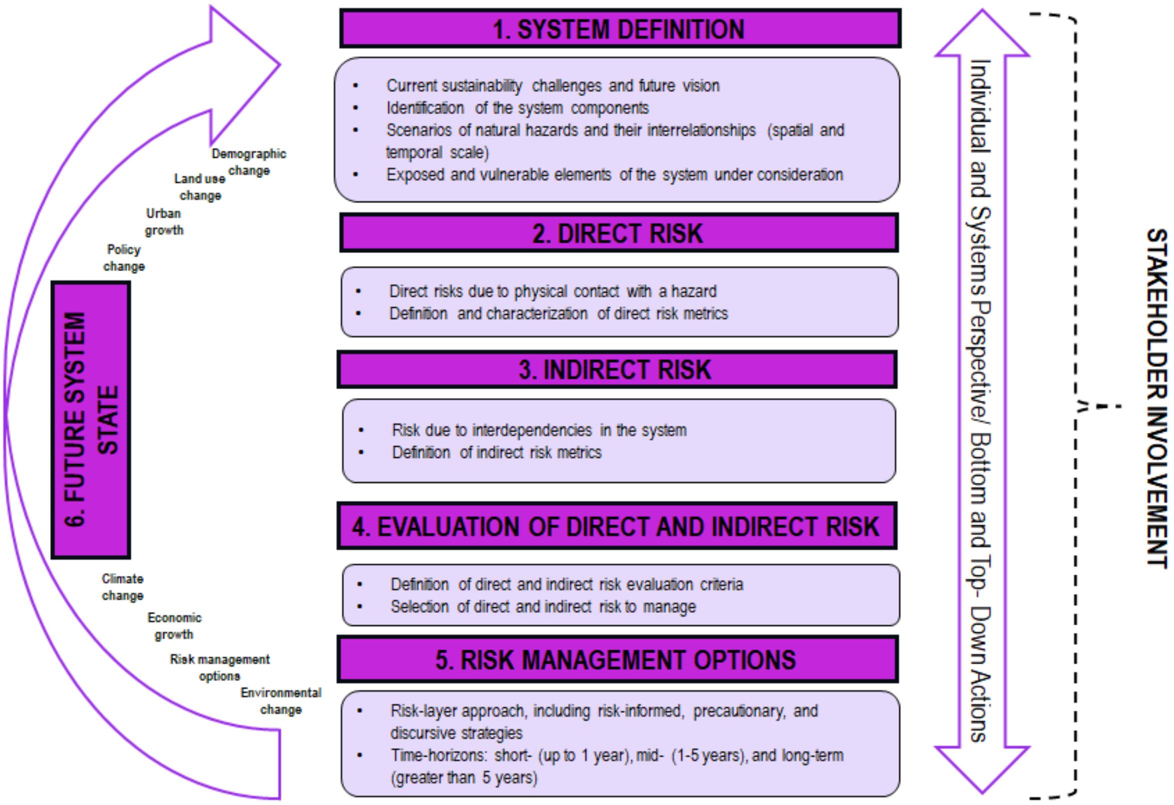

Figure S1. The prototype version of the framework used during the expert workshop in April 2022. Related to Section 2.5.
